# Supplementary material for: A Novel Approach toward Less Invasive Multiomics Gut Analyses: a Pilot Study
Source: Microbiol Spectr. 2022 Mar 28;10(2):e02446-21. doi: 10.1128/spectrum.02446-21 (PMC9045184; doi:10.1128/spectrum.02446-21)
Supplement: SUPPLEMENTAL FILE 1 — Supplemental material. Download SPECTRUM02446-21_Supp_1_seq5.pdf, PDF file, 0.6 MB [file spectrum02446-21_supp_1_seq5.pdf]

Supplemental Table 1

Sample Method	Sequence Depth (Gbp)	Total Reads (million)	Bacterial Reads (%)
Biopsy	2.04±0.47	14.5±7.2	26.5±11.5
Cytology Brush	4.22±3.03	41.7±31.9	59.1±23.9*

Mean number ± SD of gigabase pairs sequenced (Gbp), sequenced reads (in millions) after removal of poor quality reads, and bacterial percentage of reads following metatransriptomics sequencing comparing pinch biopsy and cytology brush methods. N=5 samples per group. *, $P<0.05$ as determined by Student's t-test comparing biopsy to cytology brush.

Supplemental Table 2

Class	Mean ± SEM (%)		P value
	**Biopsy**	**Cytology Brush**	
Methanobacteria	1.08 ± 2.42	1.66 ± 3.71	0.778
Actinobacteria	56.11 ± 21.16	22.26 ± 14.88	**0.019**
Bacteroidia	2.21 ± 4.95	11.58 ± 14.89	0.219
Bacilli	29.35 ± 5.43	10.82 ± 11.33	**0.011**
Clostridia	4.11 ± 7.32	47.71 ± 10.84	**0.00007***
Negativicutes	0.80 ± 1.78	1.28 ± 2.85	0.757
Betaproteobacteria	0.03 ± 0.05	1.28 ± 2.55	0.306
Deltaproteobacteria	0.00 ± 0.00	0.65 ± 1.45	0.346
Epsilonproteobacteria	0.00 ± 0.00	0.07 ± 0.16	0.346
Gammaproteobacteria	6.31 ± 7.00	2.68 ± 2.32	0.304

Top 10 bacterial classes identified as different between the two collection methods. Data shown as mean ± SEM. Three classes noted to be significantly different with p-value <0.05 and one with FDR <0.05 as indicated by the asterisk.

Supplemental Table 3

Species	Mean ± SEM (%)		P-value
	Biopsy	Cytology Brush	
Faecalibacterium prausnitzii	2.27 ± 3.36	17.37 ± 11.62	**0.023**
Finegoldia magna	0.00 ± 0.00	12.49 ± 19.77	0.195
Methanobrevibacter unclassified	1.08 ± 2.42	2.11 ± 4.72	0.676
Parabacteroides unclassified	1.55 ± 3.46	8.52 ± 11.94	0.246
Propionibacterium acnes	53.2 ± 18.44	13.76 ± 12.17	**0.004***
Pseudomonas unclassified	5.13 ± 6.44	0.93 ± 2.07	0.202
Ruminococcus lactaris	0.01 ± 0.02	3.27 ± 3.02	**0.042**
Ruminococcus torques	1.72 ± 3.84	12.22 ± 14.01	0.145
Streptococcus thermophilus	32.14 ± 4.69	13.54 ± 15.18	**0.031**
Subdoligranulum unclassified	0.13 ± 0.29	4.35 ± 5.54	0.127
Others	2.68 ± 2.09	11.44 ± 7.71	**0.039**

Top 10 bacterial species identified as different between the two collection methods. Data shown as mean ± SEM. Four species noted to be significantly different with p-value <0.05 and one with FDR <0.05 as indicated by the asterisk.

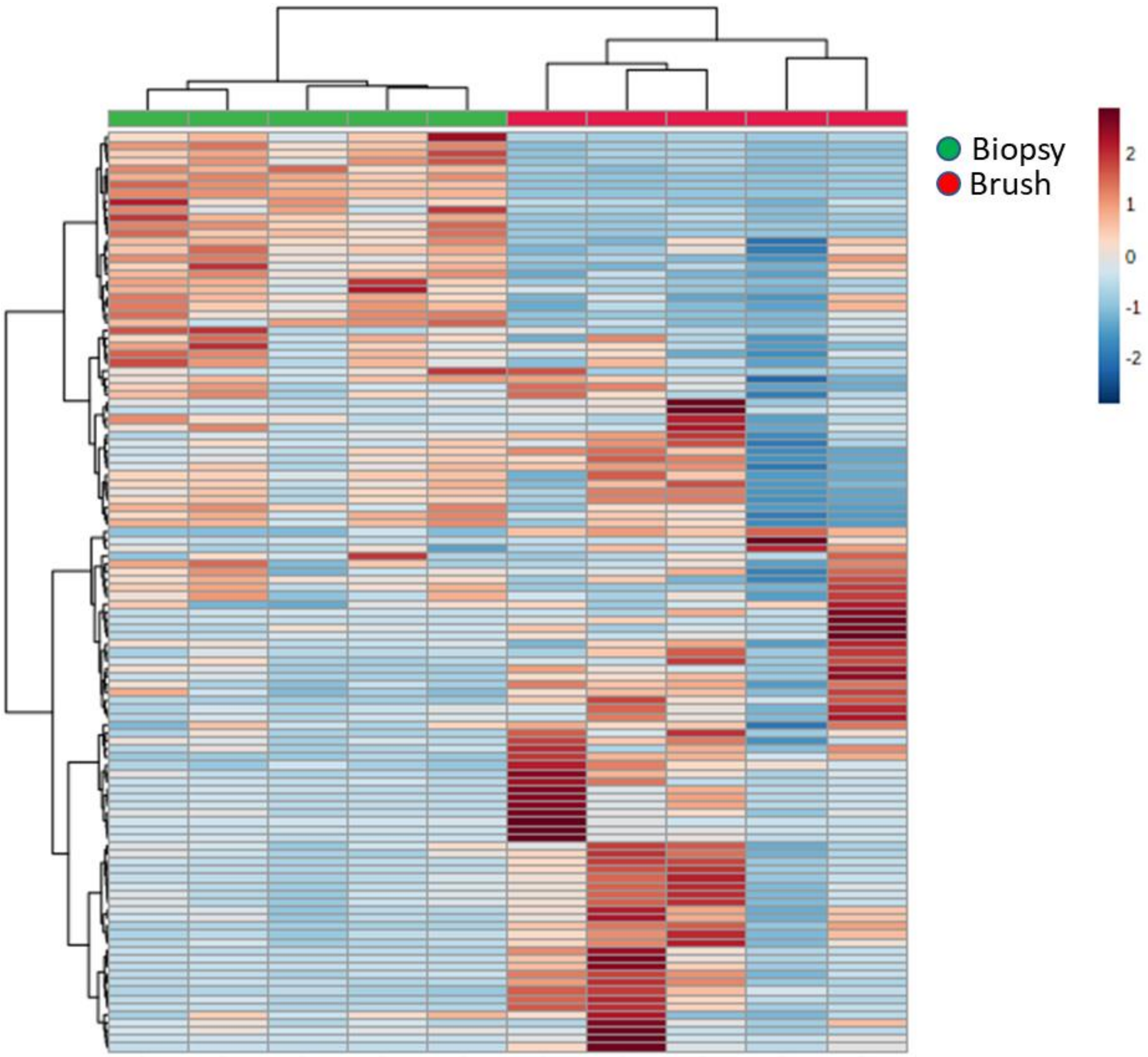

Supplemental Figure 1. Heat map demonstrating all 114 metabolites comparing the pinch biopsy versus cytology brush collection methods.

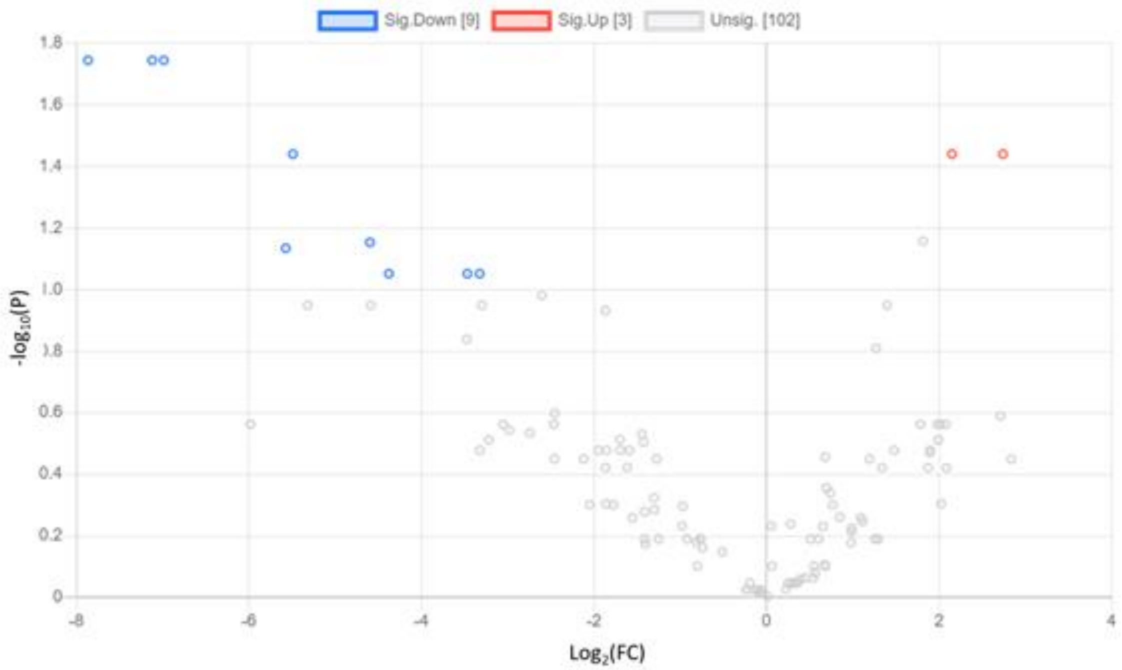

Supplemental Figure 2. Volcano plot demonstrating 114 metabolites identified as significantly up (red), down (blue), or unchanged (gray) comparing the pinch biopsy versus cytology brush collection method. Significance determined by fold change >2.0 and Wilcoxon signed-rank test with FDR adjusted p-value <0.1.

Supplemental Table 4

Metabolite	Log_2(FC)	P-value (FDR adjusted)
D-Glucono-1-5-lactone 6-phosphate*	-7.86	0.018
Cys-Gly	-7.12	0.018
alpha-D-Glucosamine 1-phosphate*	-6.98	0.018
5-hydroxytryptophan†	-5.45	0.036
2-Hydroxyglutarate/Citramalate	2.74	0.036
Itaconate	2.15	0.036
L-cysteine	-4.59	0.070
D-Erythrose 4-phosphate	-5.57	0.073
N5-Methyl-L-glutamine	-4.38	0.089
N-Acetyl-L-citrulline	-3.47	0.089
CMP	-3.32	0.089

Metabolites identified as significant (fold change, FC >2.0 and paired-t-test with FDR adjusted p-value <0.1) for comparison between pinch biopsy and cytology brush collection method.
